# Photosynthetic functions of *Synechococcus* in the ocean microbiomes of diverse salinity and seasons

**DOI:** 10.1371/journal.pone.0190266

**Published:** 2018-01-02

**Authors:** Yihwan Kim, Jehyun Jeon, Min Seok Kwak, Gwang Hoon Kim, InSong Koh, Mina Rho

**Affiliations:** 1 Department of Computer Science and Engineering, Hanyang University, Seoul, Korea; 2 Department of Biology, Kongju National University, Kongju, Korea; 3 Department of Biomedical Informatics, Hanyang University, Seoul, Korea; University of North Texas, UNITED STATES

## Abstract

*Synechococcus* is an important photosynthetic picoplankton in the temperate to tropical oceans. As a photosynthetic bacterium, *Synechococcus* has an efficient mechanism to adapt to the changes in salinity and light intensity. The analysis of the distributions and functions of such microorganisms in the ever changing river mouth environment, where freshwater and seawater mix, should help better understand their roles in the ecosystem. Toward this objective, we have collected and sequenced the ocean microbiome in the river mouth of Kwangyang Bay, Korea, as a function of salinity and temperature. In conjunction with comparative genomics approaches using the sequenced genomes of a wide phylogeny of *Synechococcus*, the ocean microbiome was analyzed in terms of their composition and clade-specific functions. The results showed significant differences in the compositions of *Synechococcus* sampled in different seasons. The photosynthetic functions in such enhanced *Synechococcus* strains were also observed in the microbiomes in summer, which is significantly different from those in other seasons.

## Introduction

Microbes adapt to their environments to play crucial roles in the ecosystems. In the marine environment, cyanobacteria are major contributors for the carbon and nitrogen cycles, carrying out about 25% of the total carbon fixation in the ocean [1]. In addition, cyanobacteria are known as the only bacteria that perform oxygenic photosynthesis [2]. Such crucial roles would inevitably be affected by the global warming, and result in the changes of marine microbial communities. Due to their ecological importance and the benefit of small genome size, many genomes have been sequenced and studied in diverse genera in cyanobacteria [3].

*Synechococcus* belongs to *Cyanobacteria*, and is one of the most important components of photosynthetic picoplankton in the temperate to tropical oceans. It is widespread throughout the global oceanic surface and freshwater environment with different temperature, thermal amplitude, light, and nutrients [1, 4–8]. Compared with *Prochlorococcus*, which is another major constituent in cyanobacteria, *Synechococcus* shows a wider geographical distribution [1]. As a photosynthetic bacterium, *Synechococcus* also has an efficient mechanism to adapt to the changes in salinity and light intensity [9, 10]. Therefore, analysis of the distributions and functions of such microorganisms in the ever changing river mouth environment, where freshwater and seawater mix, should help better understand their roles in the ecosystem [11, 12].

With recent advances in the sequencing technology and computational methods, more comprehensive genomic approaches are applied to survey the compositions and functions of the entire microbial community. The pioneering ocean microbiome project, Global Ocean Sampling (GOS) Project, started in early 2000 [13]. Since then, many ocean microbiome projects have unveiled the global landscape of the bacterial distributions and their functions in the marine environments [14–16]. Such studies suggest that the ocean microbiomes are significantly diverse in terms of depth, temperature, and nutrient. For example, the Tara Oceans Project showed a significant correlation among the species, functional richness, and water depth [17]. In particular, photosynthetic cyanobacteria such as *Prochlorococcus* and *Synechococcus* were observed in all mesopelagic samples, constituting about 1% of the composition. However, there are few metagenomic studies on a group of photosynthetic algae in the river mouth environment.

In this study, we have profiled the distribution of *Synechococcus* collected in the river mouth of Kwangyang Bay, Korea, as a function of salinity and temperature. Five collection sites were selected from the freshwater in Seomjingang river to the open sea of Kwangyang Bay. In an effort to identify the contributors of *Synechococcus* in microhabitats in detail, we found close *Synechococcus* strains and analyzed their clade-specific functions in higher resolution. In conjunction with comparative genomics approaches [18–20] using the sequenced genomes in a wide phylogeny of *Synechococcus*, the Korean ocean microbiome data was analyzed in terms of the composition and functions. In addition, the major lineages of the *Synechococcus* strains in the microbiome and their phylogenetic characteristics were investigated as well.

## Results

### *Synechococcus* composition in the marine microbiome

**Fig 1:**
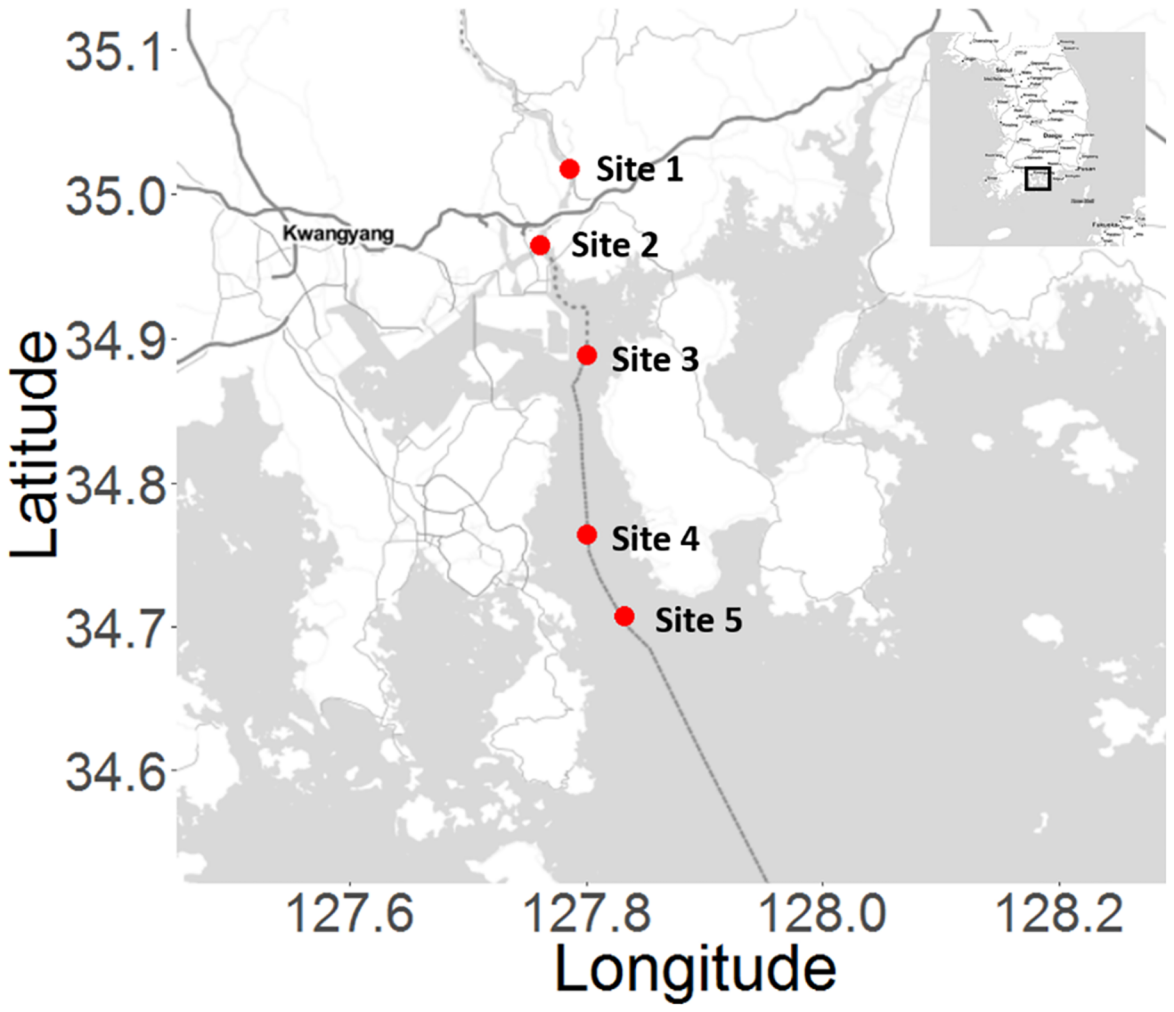
Microbiome collection sites in Kwangyang bay, Korea.

The distribution of *Synechococcus* was investigated as a function of the temperature and salinity by using a total of 15 ocean samples that were collected from five different sites at three different time points. The salinity of the samples increases from site 1 to site 5. The temperature ranges from 7 to 8°C for the winter samples; from 16 to 17°C for the spring samples; from 24 to 26°C for the summer samples (Fig 1, Table 1). The seasonal changes in the bacterial composition of five collection sites were profiled using marker genes (S1 Fig). Interestingly, *Synechococcus* blooms were observed only in the samples collected in summer, not in winter or spring.

**Table 1:** List of ocean microbiomes.

*Sample*^a^	*Description*	*Sampling date*	*Latitude*	*Longitude*	*Sal(ppt)*	*Temp*.*(°C)*	*Size(Gbps)*
SKY1B1502	Site 1	02/24/2015	35°01′05″N	127°47′07″E	8.5	8	9.0*2
SKY2B1502	Site 2	02/24/2015	34°57′55″N	127°45′40″E	26	8	8.8*2
SKY3B1502	Site 3	02/24/2015	34°53′20″N	127°48′00″E	35	7	18*2
SKY4B1502	Site 4	02/25/2015	34°45′50″N	127°48′00″E	35	8.2	11*2
SKY5B1502	Site 5	02/25/2015	34°42′28″N	127°49′56″E	35	8.2	11*2
SKY1B1505	Site 1	05/07/2015	35°01′05″N	127°47′07″E	2.5	17	33*2
SKY2B1505	Site 2	05/07/2015	34°57′55″N	127°45′40″E	21	17	37*2
SKY3B1505	Site 3	05/07/2015	34°53′20″N	127°48′00″E	31	17	33*2
SKY4B1505	Site 4	05/08/2015	34°45′50″N	127°48′00″E	32	16	33*2
SKY5B1505	Site 5	05/08/2015	34°42′28″N	127°49′56″E	34.5	16	27*2
SKY1B1508	Site 1	08/12/2015	35°01′05″N	127°47′07″E	23	26	35*2
SKY2B1508	Site 2	08/12/2015	34°57′55″N	127°45′40″E	29	26	35*2
SKY3B1508	Site 3	08/12/2015	34°53′20″N	127°48′00″E	35	24	33*2
SKY4B1508	Site 4	08/12/2015	34°45′50″N	127°48′00″E	34	24	36*2
SKY5B1508	Site 5	08/12/2015	34°42′28″N	127°49‘56″E	35	24	35*2

^a^ The first three letters of the sample name represent “*Sea of Kwangyang*”. The fourth and fifth letters (1B, 2B, 3B, 4B, and 5B) represent the specific site. The last four digit numbers represent sampling date (year and month, YYMM): 1502 for winter, 1505 for spring, and 1508 for summer.

**Fig 2:**
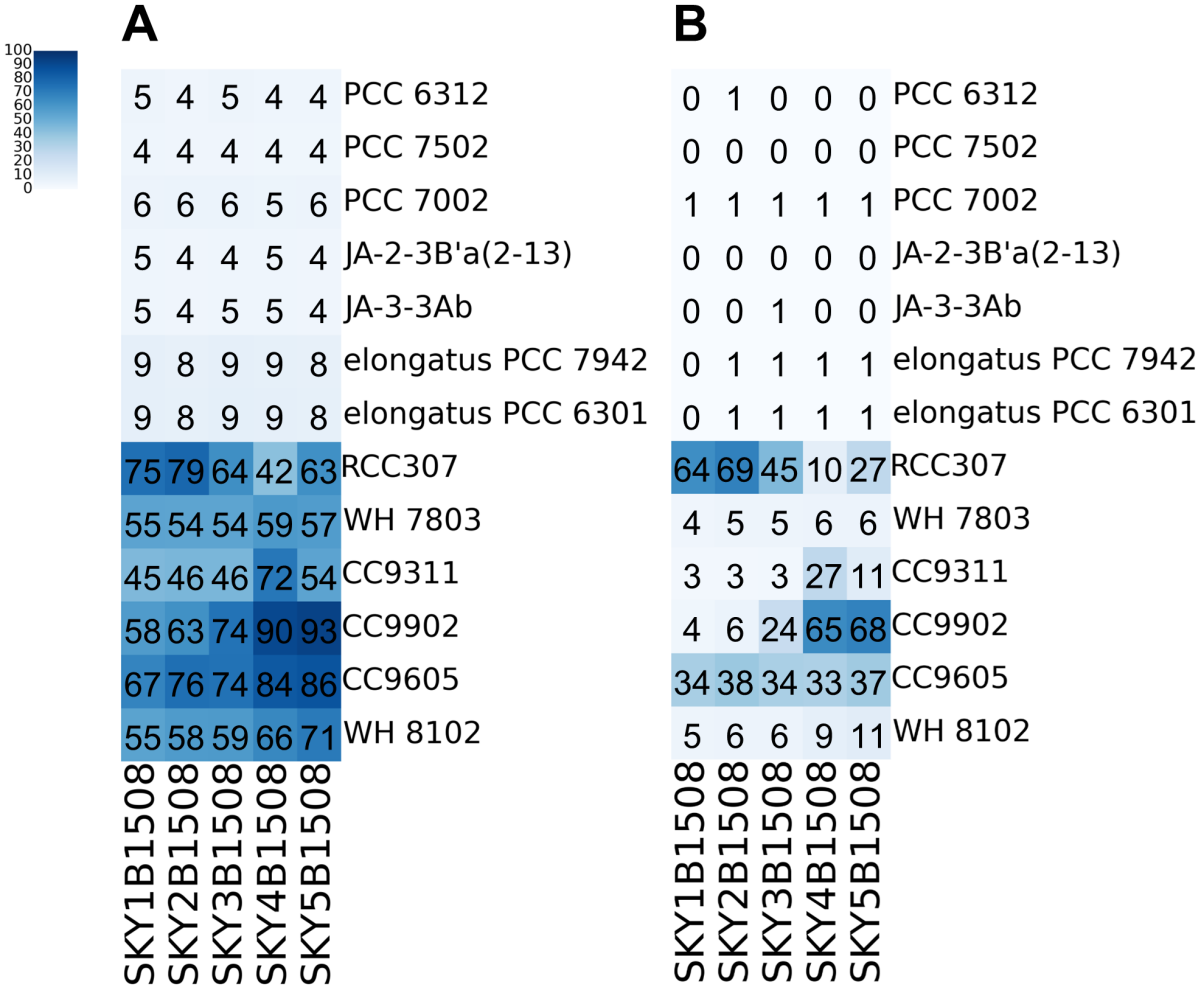
Coverage of entire genes of the *Synechococcus* strains in each microbiome sample. **(A)** Gene coverage of each strain by using CD-HIT clustering with 70% protein similarity. **(B)** Gene coverage of each strain with 95% protein similarity. The values in cells are the ratio of genes clustered (%) in each strain.

The distribution of *Synechococcus* strains was further investigated by using all *Synechococcus* genes in the 13 *Synechococcus* complete genomes (S1 Table). The entire set of genes were clustered together with those predicted from the five microbiomes of the summer. When the clustering threshold of 70% sequence similarity was applied, a total of 75,215 genes in the ocean microbiomes were clustered with most of the genes in six strains (Fig 2A). Different *Synechococcus* strains dominated in freshwater (RCC307 in site 1 and 2) and seawater (CC9902 and CC9605 in site 4 and 5) environments. When the clustering threshold was increased to 95%, this pattern became more evident (Fig 2B). For the strains other than RCC307, CC9902 and CC9605, the proportion of the genes that were clustered with each metagenome was mostly smaller than 10% of each strain, which might be the housekeeping genes. With more stringent clustering threshold of 95%, more exclusive clustering was observed (Fig 2B). This finding implies that the genes in the microbiome are highly homologous with six *Synechococcus* strains, not with the other seven strains (Fig 2B).

Interestingly, two samples collected in river mouth (SKY1B1508) and water front (SKY2B1508) are enriched with RCC307, whereas the other two samples collected in open sea (SKY4B1508 and SKY5B1508) are enriched with CC9902 (Fig 2B). As freshwater and seawater mixes in river mouth (Site 1) and water front (Site 2), the salinity varied in the range of 2.5–29 ppt depending on sampling season and time (Table 1). In details, when the clustering threshold of 95% sequence similarity was applied, 64% and 69% of the genes in RCC307 were clustered with the genes in the SKY1B1508 and SKY2B1508, respectively, with the average similarity of 99.26%. Samples collected from sites in the open sea (Site 4 and 5, salinity >30 ppt) showed different enrichment patterns. About 65% and 68% of the genes in CC9902 were clustered with the genes in SKY4B1508 and SKY5B1508, respectively, with the average similarity of 98.34%. The sequence similarities against two strains, RCC307 and CC9902, are high enough to suggest that the two groups of samples with lower salinity and higher salinity are enriched with two different taxa close to *Synechococcus* RCC307 and CC9902, respectively. These results suggest that the populations of *Synechococcus* changed at the border of waterfront although there was no barrier but the salinity difference. A similar pattern was observed in the previous study, which showed that CC9902 is positively correlated with salinity in the surface layer of the ocean [21]. In addition, the strain CC9902 was also observed in the location of similar latitude as the samples in this study, considering the observation that Clades I and IV are enhanced at the latitudes above 30°N and below 30°S [8]. Notably, the strain CC9902 was observed in the summer sample (24°C for Site 4 and 5), but was not observed in the winter or spring samples (8.2 and 16°C, respectively).

**Fig 3:**
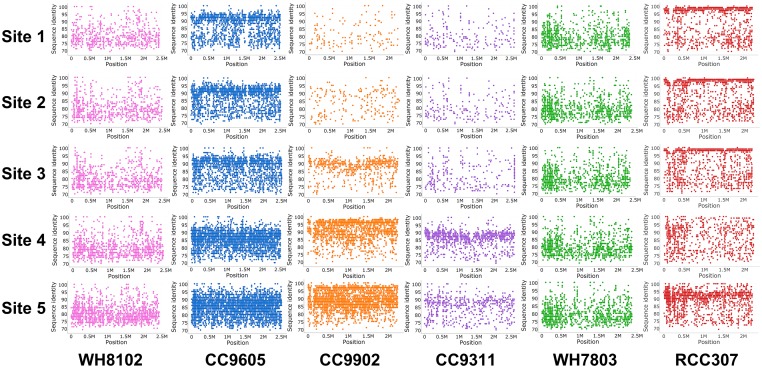
Homology of contigs assembled from the microbiome against the *Synechococcus* strains. Each dot in scatter plot indicates the contig at the specific genome position (x-axis) with the sequence similarity against the reference genome (y-axis).

In order to find the homology of contigs that were assembled from the ocean microbiome, BLAST search [22] was applied against six complete genomes of *Synechococcus* as a reference. With 95% sequence similarity, RCC307 was covered in the sites 1, 2 and 3, whereas CC9902 was covered in the sites 4 and 5 (Fig 3). A total of 680, 693, and 581 contigs that cover 52.59% of genomes with higher than 95% of average similarity were assembled from the sites 1, 2, and 3. A total of 582 and 630 contigs that cover 46.47% of genomes with higher than 95% of average similarity were assembled from the sites 4 and 5.

### Phylogenetic analysis of *Synechococcus* strains

A phylogenetic analysis was conducted on 13 complete *Synechococcus* genomes. In conjunction with comparative genomics approaches, all genes were clustered to find the orthologous genes in the *Synechococcus* strains. The genes are classified into three different types of genes: core genes that are shared by all strains; dispensable genes that are shared by some of the strains; strain-specific genes that are unique to a strain.

**Fig 4:**
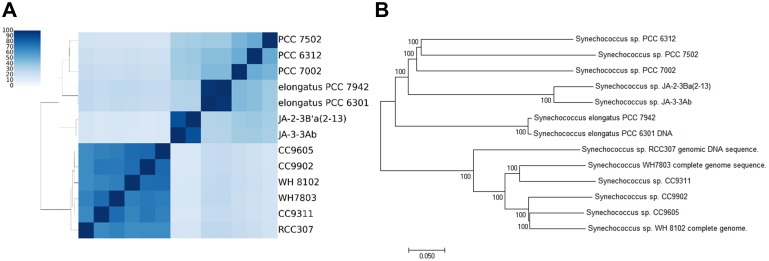
Phylogenetic tree of thirteen *Synechococcus* strains. **(A)** Heatamp of Jaccard index using dispensable genes. **(B)** Phylogeny using concatenated alignments of core genes.

The phylogenetic tree and clustering were conducted by using two different approaches: multilocus sequence tags (MLST) using core genes; Jaccard index using dispensable genes. Two distinctive groups were observed in both approaches: one group of strains that are enriched in the samples, and the other group of strains that are not enriched in the samples. Seven *Synechococcus* strains (PCC7502, PCC6312, PCC7002, PCC 7942, PCC6301, JA-2, and Ja-3) were clustered together, whereas the other six *Synechococcus* strains (CC9605, CC9902, WH8102, WH7803, CC9311, and RCC307) were clustered together (Fig 4).

This observation is also consistent with recent studies that suggested the division of two separate lineages, *Synechococcus* and *Parasynechococcus* [23]. The new genus *Parasynechococcus* contains the strains CC9605, CC9902, WH8102, WH7803, CC9311, and RCC307, which show high homology with the genes in the ocean microbiome. Among *Parasynechococcus* strains, RCC307 and CC9902 build two separate lineages in the phylogenetic tree with the bootstrapping value of 100 (Fig 4B). In particular, two lineages were observed exclusively in the environments of different salinity: RCC307 enriched in lower salinity; CC9902 enriched in higher salinity (Fig 4). For the third lineage that consists of WH7803 and CC9311, a limited number of genes show sequence homology with the genes in the samples.

### Phycobilisomes in *Synechococcus*

Given the important role of *Synechococcus* as a photosynthetic organism in the cyanobacterium, a comparative analysis was performed for the genes that are associated with the three components in phycobilisomes. Phycobilisomes are the major light-harvesting antennas, which are anchored to the thylakoid membranes. Phycobilisomes consist of three components, allophycocyanin (AP), phycocyanin (PC), and phycoerythrin (PE), and have different structures depending on the pigment composition. *Parasynechococcus* strains have phycoerythrobilin (PEB) and phycourobilin (PUB), which are more effective in light absorption and adaptation to the changes in light quantity [24, 25]. The results of this study revealed significantly differential gene sets that exist only in *Parasynechococcus*, but not in *Synechococcus*.

**Fig 5:**
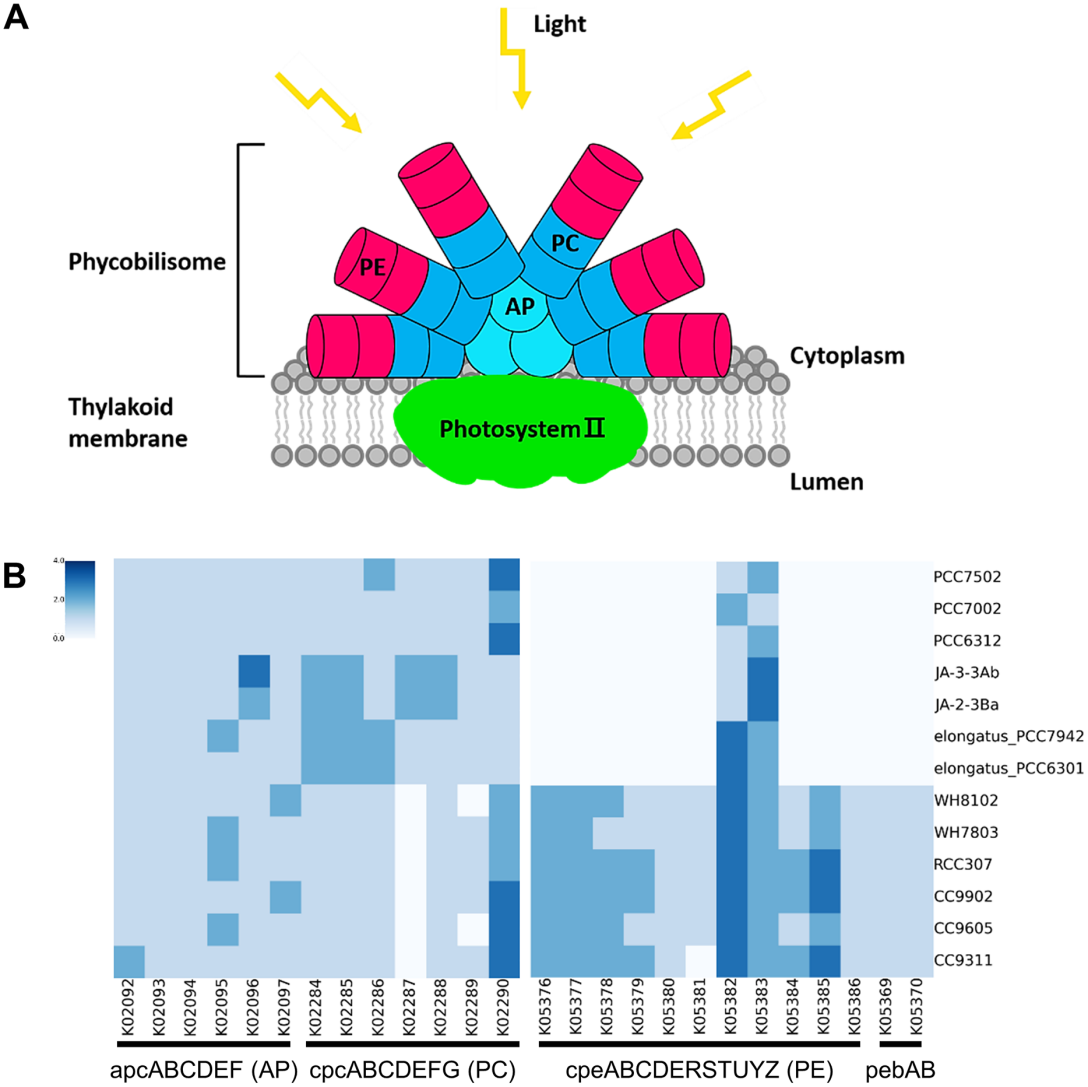
Phycobilisome gene sets in *Synechococcus*. **(A)** Phycobilisome complex. **(B)** Heatmap of gene sets involved in forming phycobilisome complex.

The phycobilisome-associated genes were screened from the 13 *Synechococcus* strains. A total of 26 KO genes that are related with 6 allophycocyanin, 7 phycocyanin, 11 phycoerythrin, and 2 phycoerythrobilin were screened to find the existence of such specific functions. All six allophycocyanin genes and most of the seven phycocyanin genes, except cpcD and cpcF, were observed in all 13 strains (Fig 5). Notably, cpcD does not exist in any of the *Parasynechococcus* strains. Since allophycocyanin and phycocyanin belong to the phycobiliprotein family, they play crucial roles as accessory pigments to chlorophyll in these photosynthetic *Synechococcus*.

Interestingly, phycoerythrin genes showed a significantly different distribution between two groups of *Synechococcus* strains. Among 13 phycoerythrin genes, 11 genes exist only in the *Parasynechococcus* strains, not in the S*ynechococcus* strains. Only two genes of cpeS and cpeT exist in all 13 strains. This observation could explain the fact that the *Parasynechococcus* strains in summer samples are enriched with the effective functions for light absorption and adaptation to changes in the light intensity.

### RecBCD operon in *Synechococcus*

**Fig 6:**
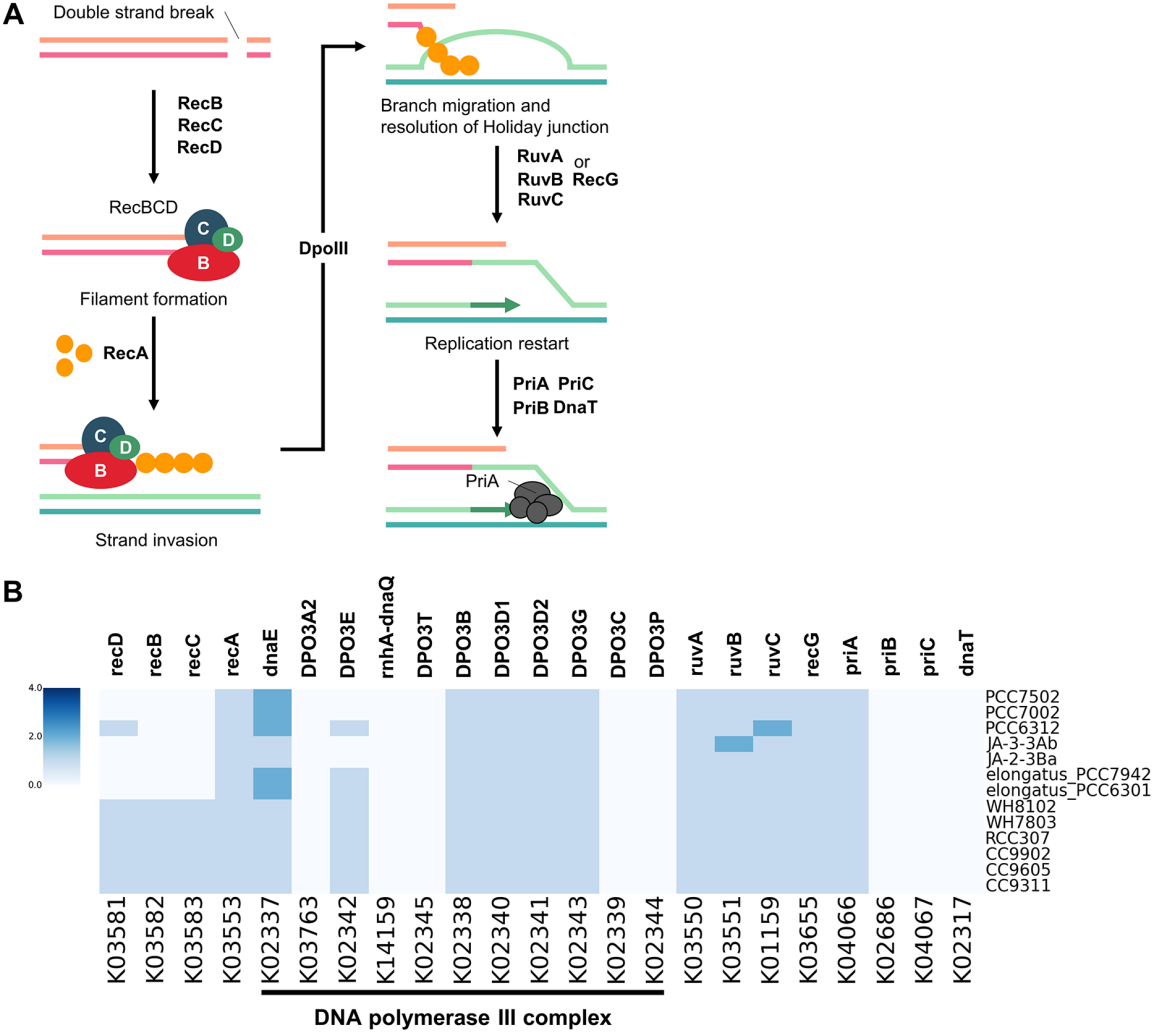
RecBCD operon in *Synechococcus*. **(A)** RecBC pathway of KEGG. **(B)** Heatmap of KEGG orthologs involved in the pathway.

RecBCD genes encode an enzyme complex that initiates homologous recombination processes that repair double-strand breaks in eubacteria [26–28]. In our KEGG analysis, these recBCD genes are only encoded in *Parasynechococcus*. Especially, KEGG orthologs from homologous recombination pathway (K03581, K03582 and K03583) were observed only in *Parasynechococcus* strains, whereas some *Synechococcus* have KEGG ortholog K01144 related to the recBCD operon (Fig 6).

Distribution of the most proteins involved in the subsequent processes of homologous recombination did not show the difference between *Parasynechococcus* and *Synechococcus*. A previous study on the comparative analysis of homologous recombination systems found that *Synechococcus* WH8102 and *Prochlorococcus marinus* strains are the only genomes having the recBCD gene set [29]. Our observation is consistent with this fact. Our results also suggest that the living conditions of *Parasynechococcus*, in which the DNA is prone to photochemical damage [30], might have forced *Parasynechococcus* to have the recBCD gene set.

The heatmap showed that there are five genes (DPO3A2, rnhA-dnaQ, DPO3T, DPO3C, and DPO3P) that do not exist in any of the *Synechococcus* strains (Fig 6B). Such genes are involved in constructing the DNA polymerase III complex. In addition, there are also three missing orthologs: priB, priC, and dnaT. Lastly, there is an ortholog showing some differences, except for recBCD operon, between the two clades. The DPO3E, which is a DNA polymerase III subunit epsilon, is encoded only in *S*. *elongatus*, *Synechococcus* PCC6312, and all *Parasynechococcus* strains known.

### Phycobilisome pathway in the marine microbiome

We observed that *Synechococcus* is one of the major constituents in the summer samples, and exists differentially among the samples of different temperatures. In particular, three strains, *CC9605*, *CC9902* and *RCC307*, in different lineages of marine *Synechococcus* were inferred as very close strains in the summer samples (Fig 2). From this observation, we hypothesize that the photosynthesis functions are significantly enhanced in the summer samples. Indeed, our metagenome analysis fully confirmed this postulation. In order to investigate the light-harvesting functions in different marine environments, the phycobilisome pathway was screened from the marine microbiome data at different salinity and temperature.

**Fig 7:**
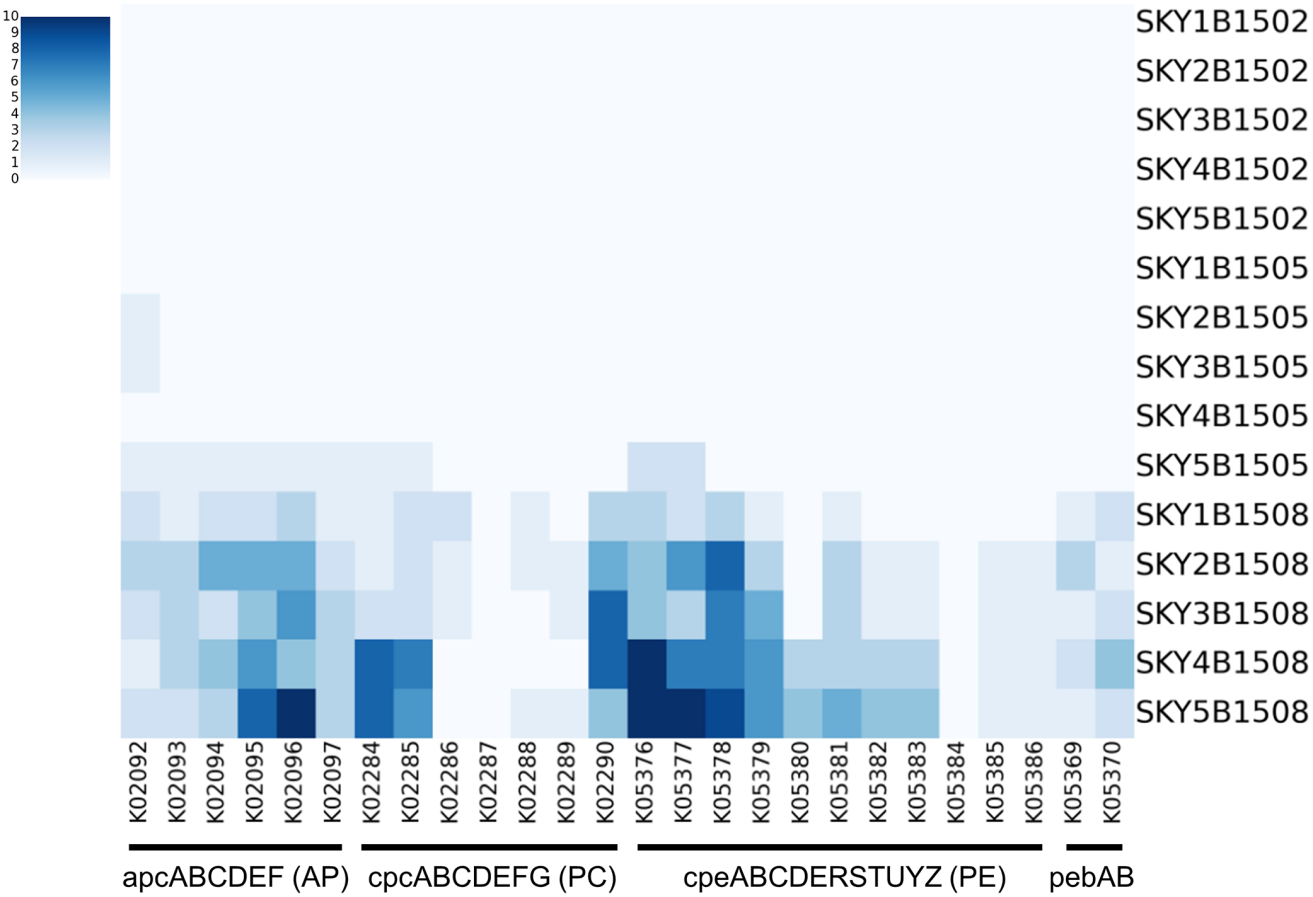
Phycobilisome gene sets in the ocean microbiomes. Heatmap of gene sets involved in forming phycobilisome complex. SKY is abbreviation of Sea of Kwangyang bay; 1B, 2B, 3B, 4B, and 5B represent the sampling sites in Fig 1; 1502, 1505, and 1508 indicate the sampling date (YYMM).

The homology search against 489 genes that belong to 26 phycobilisome-related KOs revealed that light-harvesting functions are significantly enhanced in the summer samples at different salinity (Fig 7). Notably, cpcD (phycocyanin linker protein, K02287) and cpeU (billin biosynthesis protein, K05384) were not observed in any of the summer samples. A similar result was observed when the phycobilisome-related genes were screened from 13 *Synechococcus* strains; CpcD gene was not found in any of the *Synechococcus* strains. Notably, previous study also reported that cpcD does not exist in many *Synechococcus* strains including RCC307 and CC9902 [24]. Overall, phycobilisome-related genes are widely distributed in the summer samples, but not in most of the winter or spring samples. Most of the phycobilisome-related genes are more enriched in site 4 and 5 of higher salinity. In particular, phycoerythrin genes are more enriched in site 4 and 5 (Fig 7).

Notably, no phycobilisome-related functions were found in five winter samples of different salinity. In the spring samples, most of the phycobilisome-related functions were not found; apcA genes (allophycocyanin alpha subunit, K02092) were observed in the site 2, 3, and 5 of the spring samples; all apc (allophycocyanin alpha subunit, K02092, K02093, K02094, K02095, K02096, and K02097), cpcA (phycoerythrin class chain, K05376), and cpeB (phycoerythrin class beta chain, K05377) genes were found in the site 5 of the spring sample. This might be due to the homology of such genes in other strains that are close to *Synechococcus* strains.

## Discussion

*Synechococcus* is an important photosynthetic picoplankton in the temperate to tropical oceans. As a photosynthetic bacterium, *Synechococcus* is known to have an efficient mechanism to adapt to the changes in salinity and light intensity. In this study, we investigated ocean microbiomes of Kwangyang Bay where freshwater and seawater mix. A total of fifteen ocean microbiomes were obtained from five sites of different salinity in three different seasons. The analysis revealed significant differences in the composition of *Synechococcus* strains and their photosynthetic functions in the freshwater to open sea of Kwangyang Bay in different seasons.

In order to determine the specific lineages of *Synechococcus* strains in the microbiome, we applied comparative genomics to the analysis of microbiome. Even with the limited number of sequenced genomes available, we could successfully determine the very close lineages to the *Synechococcus* strains in the ocean microbiome. Two distinctive lineages are associated with two groups of samples in different salinity.

Marine picocyanobacteria *Synechococcus* species are the most abundant and widespread primary producers in the ocean. It has been reported that *Synechococcus* partition the ocean into four distinct regimes that are distinguished by the temperature, macronutrients (nitrate and phosphate), and iron availability [8]. The results from our metagenome analysis showed that the salinity difference works as a barrier between terrestrial and oceanic species of *Synechococcus*. Although many *Synechococcus* can survive wide range of salinity, the enriched population was clearly different across the waterfront where freshwater mixes with seawater, suggesting that salinity should be considered as an environmental factor that partitions *Synechococcus* distribution.

Temperature is another important environment factor that could affect the occurrence of *Synechococcus* strains. In particular, Clades I and IV are enhanced at cold temperature (latitudes above 30°N and below 30°S), compared with the Clades II and III (latitudes between 30°N and 30°S) [8]. In our study, the strain CC9902 of Clades IV was observed in the summer samples (24°C), not in winter (8.2°C) or spring samples (16°C). The strain CC9902 is enriched in the environment of slightly higher temperature (24°C), compared with the previous study conducted at 10–20°C [8].

*Synechococcus* plays an important role as a photosynthetic organism in cyanobacteria. As shown in the comparative analysis of sequenced genomes, phycobilisome-pathway was observed only in marine *Synechococcus* strains such as RCC307, CC9902 and CC960, but not in other lineages. The photosynthetic functions of such enhanced *Synechococcus* strains are observed in the microbiomes in summer, which is significantly different from those in other seasons. The analysis of the microbiome in different seasons also showed such distinctions of the phycobilisome pathway in different seasons. It implies that salinity and temperature are important environmental factors for the composition and function of *Synechococcus* in the ocean microbiome.

Salinity affects the growth, photosynthetic parameters, and nitrogenase activity of planktonic cyanobacteria. In addition, salinity is a main controlling factor for the blooms of N_2_-fixing cyanobacteria in estuaries [31]. Although many cyanobacteria can survive in a wide range of salinity, their growth and photosynthetic efficiency are significantly affected by the salinity [32]. When cyanobacteria are exposed to high saline conditions, the amount of phycobiliprotein eventually changes during acclimation [31]. Due to the current limitations of metagenome sequencing, it was difficult to sequence and assemble the entire set of genes involved in phycobilisome pathway in this study. It is yet to be addressed whether the absence of cpcD and cpeU genes in summer samples is related to their function.

## Materials and methods

### Sample collection and preparation

A total of 15 samples were collected from five different sites representing freshwater and seawater environment in Seomjingang river and Kwangyang Bay (Fig 1). 20 L of surface water was collected in each site and brought to the laboratory for filtering to isolate metagenomics sample. The waters were successively passed through membrane filters of 20 μm, 8 μm, 3 μm, 0.45 μm, and 0.22 μm pore size (Millipore, USA). The membranes except for 20 μm and 8 μm pore size were homogenized in liquid nitrogen, and DNA was isolated using Meta-G-Nome DNA isolation Kit (Epigentre, USA). The seasonal salinity and temperature of each collection site are summarized in Table 1. Site 1 and 2 are located in the Seomjingang River. Site 3 is the river front where fresh water and seawater mix during sub- and ebbtide. Site 4 and 5 correspond to open sea environment. All raw sequencing data described in this study is available at European Nucleotide Archive (ENA) with the accession number ERP024429.

### Preparation of shotgun metagenomic sequences

Metagenomic sequencing was carried out by Macrogen (Seoul, Korea) using HiSeq 2000 platform (Illumina, San Diego, USA). Samples were prepared according to the Illumina protocols. The quantity and quality of DNA were measured by PicoGreen and Nanodrop, respectively. For a 350 bp insert size, one microgram of genomic DNA was fragmented by using Covaris. The fragmented DNAs were blunt-ended and phosphorylated. Following end repair, the appropriate library size was selected using different ratios of the sample purification beads. A single ‘A’ was ligated to the 3′ end, and Illumina adapters were ligated to the fragments. The final ligated product was quantified by using qPCR according to the qPCR Quantification Protocol Guide, and qualified by using the Agilent Technologies 2100 Bioanalyzer (Agilent technologies, Palo Alto CA, USA). All samples were sequenced to generate 151 bp paired-end reads. The average data size is 27Gbps per sample. Low-quality reads were filtered out by using Sickle [33] (Phred quality score > = 20).

### Estimation of bacterial composition

In order to analyze the relative abundance of bacterial composition in the marine microbiome, MetaPhlAn2 was applied with the default parameter options only for the bacterial composition (i.e.—ignore_archaea,—ignore-eukaryotes, and—ignore-viruses) [34]. To investigate the conservation against putative *Synechococcus* strains in the microbiome, a homology search was conducted by using BLAST [22] against 6 *Parasynechococcus* complete genomes (CC9902, CC9311, CC9605, RCC307, WH7803, and WH8102) from the NCBI repository. In each sample, the assembled contigs over 1 kbp were searched as a query with the stringent threshold (E-value < = 1E-10 and sequence identity > = 40%), and the best hits were retained. Scatter plots were generated by using Plotly [35].

### Clustering the genes in the ocean microbiome with *Synechococcus* and *Parasynechococcus* genes

The shotgun metagenome reads were filtered and assembled by using MEGAHIT [36] with default k-mer options. Subsequently, all contigs over 1,000 bp were used to identify the proteins in the microbiome by using FragGeneScan [37]. The predicted proteins in each sample were clustered with the proteins in 13 complete reference *Synechococcus*. In order to analyze the various aspects, CD-HIT [38] clustering was conducted with two different options of protein sequence similarity and shorter sequence coverage options: 70 over 70 (i.e. 70% sequence similarity over 70% of the shorter sequence); 90 over 90; 95 over 95. Subsequently, the proportion of clustered proteins of each *Synechococcus* strain was calculated and presented in the heatmap.

### Construction of phylogenetic tree on *Synechococcus* and *Parasynechococcus*

All 13 complete genomes of *Synechococcus* strains were obtained from the NCBI ftp repository (2015 April). A detailed genomic information on the *Synechococcus* strains is summarized in S1 Table. A total of 35,769 protein sequences in *Synechococcus* were clustered using CD-HIT (with 40% of protein similarity and 40% of shorter sequence coverage) [38] to find the orthologs genes in each cluster. As a result, 566 clusters were identified as core genes, 5,206 clusters as dispensable genes, and 4,030 clusters as strain-specific genes.

Jaccard Index were calculated using 5,206 dispensable proteins of 13 *Synechococcus* strains. Bi-clustering was conducted with average linkage and Euclidean distances. The phylogeny of *Synechococcus* was constructed using all identified 566 core proteins. Multiple sequence alignment was conducted separately on proteins in each core cluster by using MUSCLE [39]; all alignments in each strain were concatenated in the same order. Subsequently, neighbor-joining trees were constructed using MEGA [40] (ver. 7.0.14) with several options (100 for bootstrapping, Poisson model for substitution model, uniform rates, and pairwise deletion for gap/missing treat).

### Functional analysis of *Synechococcus* proteins and ocean microbiomes

Functional analyses were conducted on *Synechococcus* by using KEGG database v54 [41]. Proteins in *Synechococcus* were aligned against the protein sequences in KEGG database by using BLASTp [22] (E-value < 1.0E-10). The best hit of the query was assigned based on the E-value. For multiple best hits with the same E-value, alignment with the best sequence similarity was selected. Most of the KEGG genes can be mapped to KEGG ortholog, but there are a few cases that cannot be mapped to KEGG ortholog. In this case, we choose the second alignment based on the E-value and sequence similarity.

The same KEGG orthologs of the phycobilisome complex were used both for comparative genomics of *Synechococcus* strains and for the analysis of the functions in microbiomes. BLASTP of microbiome reads were performed against proteins in KEGG database (E-value < 1E-10; sequence identity > 90%).

## Supporting information

S1 Fig Bacterial composition of 15 microbiomes. (TIF)

S1 Table List of *Synechococcus* genomes. (DOCX)
