# Supplementary figures and images for: Photosynthetic functions of Synechococcus in the ocean microbiomes of diverse salinity and seasons
Source: PLoS One. 2018 Jan 2;13(1):e0190266. doi: 10.1371/journal.pone.0190266 (PMC5749766; doi:10.1371/journal.pone.0190266)

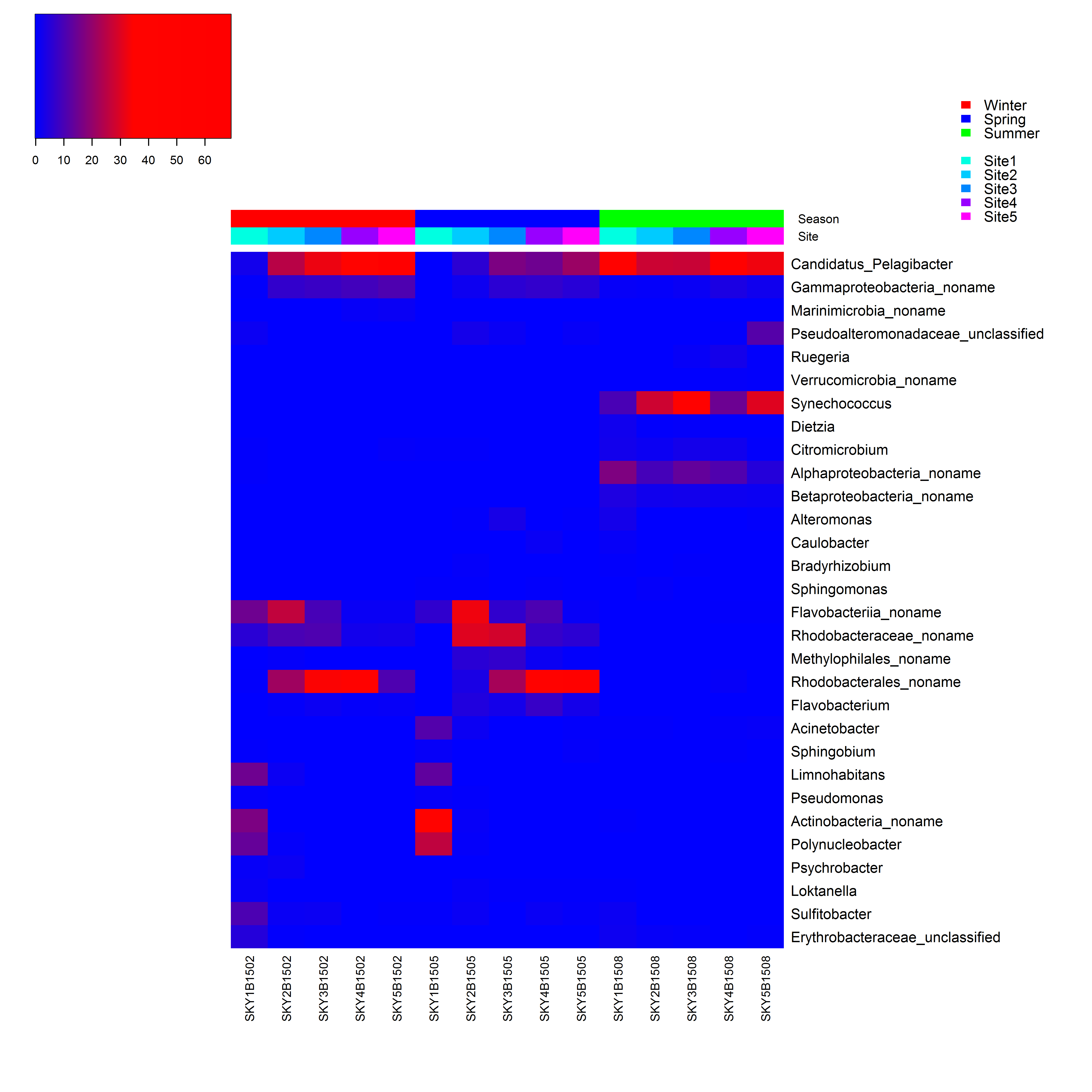

Supplement: S1 Fig — (TIF) [file pone.0190266.s001.tif]
