# Supplementary material for: Photosynthetic functions of Synechococcus in the ocean microbiomes of diverse salinity and seasons
Source: PLoS One. 2018 Jan 2;13(1):e0190266. doi: 10.1371/journal.pone.0190266 (PMC5749766; doi:10.1371/journal.pone.0190266)
Supplement: S1 Table — (DOCX) [file pone.0190266.s002.docx]

Table S1. List of *Synechococcus* genomes

| strain | NCBI accession number | # of genes | genome size (bp) | GC% |
| --- | --- | --- | --- | --- |
| Synechococcus sp. WH 8102 | NC_005070 | 2,459 | 2,434,428 | 59.41 |
| Synechococcus elongatus PCC 6301 | NC_006576 | 2,572 | 2,696,255 | 55.48 |
| Synechococcus sp. CC9902 | NC_007513 | 2,285 | 2,234,828 | 54.16 |
| Synechococcus sp. CC9605 | NC_007516 | 2,575 | 2,510,659 | 59.22 |
| Synechococcus elongatus PCC 7942 | NC_007604 | 2,651 | 2,695,903 | 55.47 |
| Synechococcus sp. JA-3-3Ab | NC_007775 | 2,576 | 2,932,766 | 60.24 |
| Synechococcus sp. JA-2-3B'a(2-13) | NC_007776 | 2,679 | 3,046,682 | 58.45 |
| Synechococcus sp. CC9311 | NC_008319 | 2,619 | 2,606,748 | 52.45 |
| Synechococcus sp. WH 7803 | NC_009481 | 2,436 | 2,366,980 | 60.24 |
| Synechococcus sp. RCC307 | NC_009482 | 2,345 | 2,224,914 | 60.84 |
| Synechococcus sp. PCC 7002 | NC_010475 | 3,110 | 3,008,047 | 49.63 |
| Synechococcus sp. PCC 6312 | NC_019680 | 3,475 | 3,697,276 | 48.52 |
| Synechococcus sp. PCC 7502 | NC_019702 | 3,342 | 3,510,253 | 40.62 |
